# Response of arboreal Collembola communities to the conversion of lowland rainforest into rubber and oil palm plantations

**DOI:** 10.1186/s12862-022-02095-6

**Published:** 2022-12-14

**Authors:** Amanda Mawan, Tamara R. Hartke, Louis Deharveng, Feng Zhang, Damayanti Buchori, Stefan Scheu, Jochen Drescher

**Affiliations:** 1 JFB Institute of Zoology and Anthropology, University of Göttingen, 37073 Göttingen, Germany; 2 L’Institut de Systématique, Évolution, Biodiversité, Muséum National d’Histoire Naturelle, 75005 Paris, France; 3 Department of Entomology, College of Plant Protection, Nanjing Agricultural University, Nanjing, 210095 China; 4 Department of Plant Protection, Faculty of Agriculture, IPB University, Bogor, 16680 Indonesia; 5 Centre of Biodiversity and Sustainable Land Use, University of Göttingen, 37077 Göttingen, Germany

**Keywords:** Arboreal arthropods, Land-use change, Oil palm, Rubber agroforestry, Springtails, EFForTS, Southeast Asia, Indonesia

## Abstract

**Background:**

In the last decades, Southeast Asia has experienced massive conversion of rainforest into rubber and oil palm monoculture plantations. The effects of this land-use change on canopy arthropods are still largely unknown. Arboreal Collembola are among the most abundant canopy arthropods in tropical forests, potentially forming a major component of the canopy food web by contributing to the decomposition of arboreal litter and being an important prey for canopy arthropod predators. We investigated abundance, richness, and community composition of, as well as the influence of a series of environmental factors on, canopy Collembola communities in four land-use systems in Jambi Province, Sumatra, Indonesia: (1) lowland rainforest, (2) jungle rubber (rubber agroforest), and monoculture plantations of (3) rubber and (4) oil palm.

**Results:**

Using canopy fogging in 32 research plots in both the dry and rainy seasons in 2013, we collected 77,104 specimens belonging to 68 (morpho) species. Generally, Collembola communities were dominated by few species including two species of the genus *Salina* (Paronellidae; 34% of total individuals) and two species of Lepidocyrtinae (Entomobryidae; 20%). The abundance of Collembola in lowland rainforest (53.4 ± 30.7 ind. m^−2^) was more than five times higher than in rubber plantations, and more than ten times higher than in oil palm plantations; abundances in jungle rubber were intermediate. Collembola species richness was highest in rainforest (18.06 ± 3.60 species) and jungle rubber (16.88 ± 2.33 species), more than twice that in rubber or oil palm. Collembola community composition was similar in rainforest and jungle rubber, but different from monoculture plantations which had similar Collembola community composition to each other. The environmental factors governing community composition differed between the land-use systems and varied between seasons.

**Conclusions:**

Overall, this is the first in-depth report on the structure of arboreal Collembola communities in lowland rainforest and agricultural replacement systems in Southeast Asia. The results highlight the potentially major consequences of land-use change for the functioning of arboreal arthropod food webs.

**Supplementary Information:**

The online version contains supplementary material available at 10.1186/s12862-022-02095-6.

## Background

Rainforests are among the oldest and most diverse ecosystems on earth, harboring two-thirds of the world's biodiversity [1]. However, in the last decades, tropical rainforests have been cleared at an alarming rate and converted into agricultural land, estate crops and plantation forests to meet the demand for food, fiber, oil, pulp and plywood, as well as other goods [2]. These transformation processes threaten biodiversity and ecosystem functions [3]. This applies in particular to Indonesia, where almost half of the rainforest area has been transformed into agricultural production systems in the last five decades. The lowlands of Sumatra lost about 50% of its former rainforest with the highest rate of deforestation in the provinces of Riau, Jambi, Bangka Belitung and South Sumatra [4–7]. The increasing rate of land-use change at the expense of rainforests led to habitat loss, fragmentation and degradation, resulting in massive species loss and altered community compositions in the converted ecosystems [8–10], thereby affecting ecosystem services and functions [11–13].

Forest canopies form a critical boundary between terrestrial ecosystems and the atmosphere [14]. The canopy of tropical rainforests represents one of the most complex habitats on earth and harbors exceptionally diverse communities of invertebrates, in particular insects [15–17]. However, most studies investigating these communities in detail have focused on arthropod taxa of rather large body size, such as cockroaches [18], beetles [19, 20], ants [21–23], homopterans [24], spiders [25, 26] and butterflies [27–29], but ignored less conspicuous taxa such as springtails (Collembola).

Detritivores, including oribatid mites (Oribatida) and Collembola, play critical roles in decomposition, nutrient cycling and plant growth [30–33], and colonize a variety of microhabitats in the canopy of trees, such as epiphytes, the bark of trees and in particular suspended soil [34–38]. Suspended (or arboreal or canopy) soil is formed from litter debris accumulating in pockets in the canopies of trees. Suspended soil may contribute significantly to nutrient cycling, particularly in the tropics [35, 39, 40]. It contains higher amounts of organic matter than most soils (approximately 75%), and these rich habitats may harbor diverse communities of micro- and mesofauna with their density exceeding that of the forest floor [38, 39, 41]. Collembola in suspended soil may reach very high densities, up to ten times that in litter and soil on the forest floor [39, 42]. Many species of mites (Acari) and Collembola in the canopy of trees are specialists, exclusively living in the canopy and not on the forest floor [43, 44].

Due to their high density, Collembola may significantly contribute to the food web of the forest canopy not only by affecting litter decomposition and canopy microorganisms, but also by serving as prey for predators [45–47]. In fact, Collembola are among the most abundant arthropods in the canopy of trees in tropical rainforests [48]. Despite the potentially important role of Collembola in arboreal habitats, community composition and the driving factors of Collembola communities in tree canopies remain poorly studied, particularly in the tropics (but see [49]). Moreover, as typical soil animals, Collembola are sensitive to low moisture conditions and high temperatures, and therefore likely respond to both seasonal fluctuations in rainfall and temperature as well as changes in canopy structure and the conversion of rainforest into plantation systems. Collembola, therefore, may serve as an environmental indicator [50, 51]. Unfortunately, knowledge on the response of arboreal Collembola to land-use change, conversion of tropical rainforest into plantations systems and seasonal changes is lacking. In this study, we present the first in-depth, species-level analysis of the structure of arboreal Collembola communities in the lowland rainforest of Jambi Province, Sumatra, Indonesia, and their response to the conversion of rainforest into jungle-rubber agroforest, and rubber and oil palm monoculture plantations in the dry and rainy season. Jungle rubber represents a low-intensity agroforest system dominated by rubber, but also contains rainforest tree species [52].

We used detailed (morpho)species level data to examine changes in the community composition of arboreal Collembola associated with the conversion of rainforest into plantation systems at the landscape scale and analyzed climatic variables to determine potential driving factors. Based on previous findings on other taxa in the same research framework [20, 22, 23, 25, 26, 29, 53–56], we tested the following hypotheses: (1) Rainforest conversion to agroforestry systems and monocultures of rubber and oil palm are associated with a decline in abundance and diversity of arboreal Collembola. (2) The arboreal Collembola community in the jungle rubber agroforest is similar to that in the rainforest. (3) The abundance and community composition of Collembola varies between seasons and this is most pronounced in monoculture plantations of rubber and oil palm due to their open canopy structure.

## Results

### Abundance and diversity

A total of 77,104 Collembola were collected, 41,940 specimens in the dry and 35,164 specimens in the rainy seasons, comprising 9 families, 19 genera and 68 (morpho) species. Paronellidae (50% of the total abundance, 25 species) and Entomobryidae (48% of the total abundance, 18 species) dominated in family-level abundance and species richness across landscapes and land-use systems. Nine of the 25 species of Paronellidae were of the genus *Salina* MacGillivray. The most abundant species in this study were *Salina* sp.01 (18% of total) and *Salina* sp.02 (16%) (Paronellidae), followed by Lepidocyrtinae sp.01 (12%) and Lepidocyrtinae sp.03 (8%) (Entomobryidae).

**Fig. 1:**
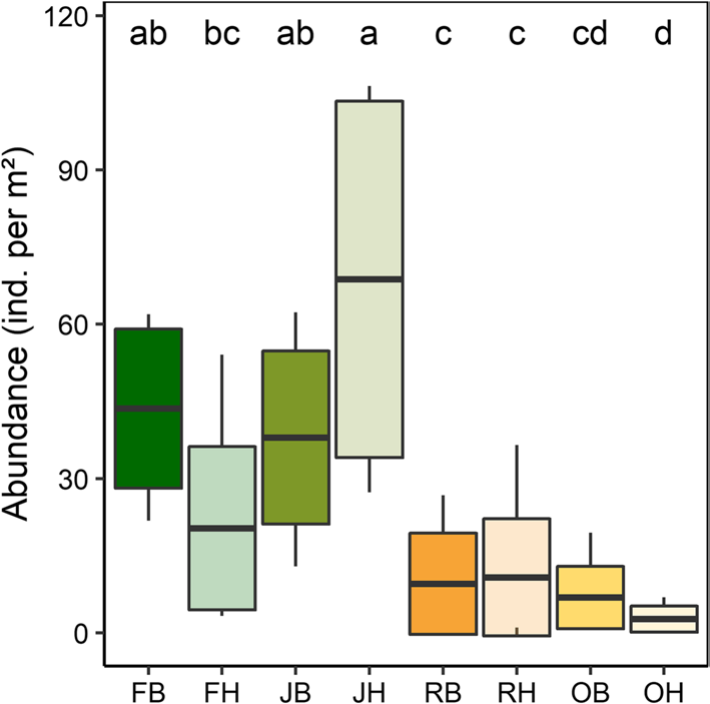
Effects of the land-use system [rainforest (**F**), jungle rubber (**J**), rubber plantations (**R**) and oil palm plantations (**O**)] and landscape [Bukit Duabelas (**B),** Harapan (**H**)] on the abundance of Collembola in tree canopies (ind. m^−2^). Bars sharing the same letter do not differ significantly, Tukey’s HSD test (p > 0.05); means ± SD (boxes) and range of minimum to maximum values (vertical line)

Generally, the abundance of Collembola in oil palm (4.78 ± 4.98 ind. m^−2^) and rubber plantations (10.17 ± 10.31 ind. m^−2^) was much lower than in jungle rubber (53.37 ± 30.71 ind. m^−2^) and rainforest (31.98 ± 19.32 ind. m^−2^), but the variation in Collembola abundance among land-use systems depended on landscape (glm; significant Land use ⨯ Landscape interaction; F_3,63_ = 6.32, p = 0.001; Fig. 1). Collembola abundance was similarly low in the two monoculture plantation systems in both the Bukit Duabelas and Harapan landscape. By contrast, abundance in the Bukit Duabelas landscape was similar in rainforest and jungle rubber, whereas in the Harapan landscape Collembola abundance in jungle rubber significantly exceeded that in rainforest. Interactions between Land use and Season (glm; F_3,63_ = 2.33, p = 0.09), Landscape and Season (glm; F_1,63_ = 0.15, p = 0.70), and Land use, Landscape and Season (glm; F_3,63_ = 0.31, p = 0.82) were not significant.

**Fig. 2:**
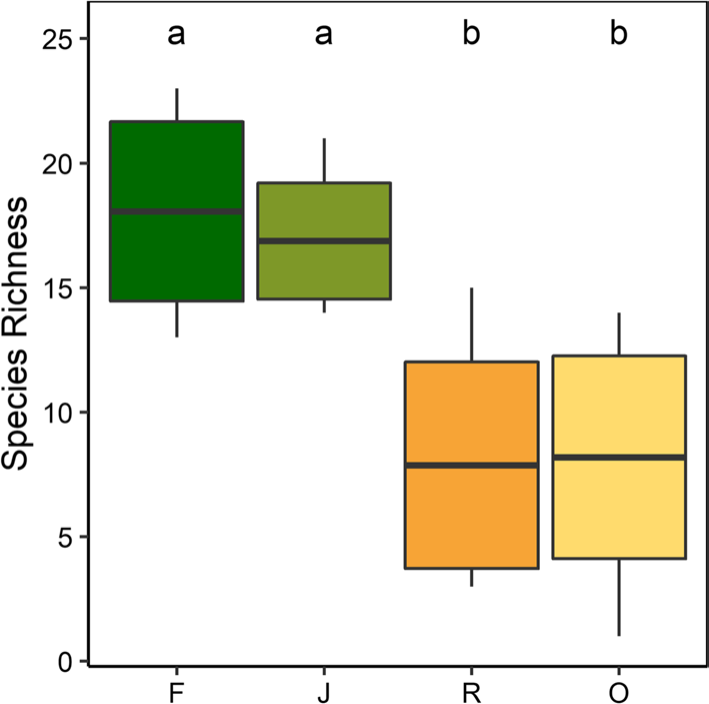
Species richness of canopy Collembola in the four land-use systems studied [rainforest (**F**), jungle rubber (**J**), rubber plantations (**R**) and oil palm plantations (**O**)]. Bars sharing the same letter do not differ significantly, Tukey’s HSD test, p > 0.05); means ± SD (boxes) and range of minimum to maximum values (vertical line). Variations with landscape and season are not shown as they did not significantly affect Collembola species richness

In addition to abundance, Collembola species richness was also significantly affected by Land use (glm; F_3,60_ = 36.71, p < 0.001); species richness in rainforest and jungle rubber was similar, and exceeded that in rubber and oil palm plantations by more than a factor of two (Fig. 2). Neither the two-way interactions between Land use and Landscape (lm; F_3,48_ = 0.65, p = 0.59), Land use and Season (lm; F_3,48_ = 0.69, p = 0.56) nor the three-way interaction between Land use, Landscape and Season were significant (lm; F_3,48_ = 0.61, p = 0.61).

Species-rank abundance curves of canopy Collembola showed a similar pattern, with rainforest and jungle rubber being similar to each other and having higher abundance and species number than rubber and oil palm plantations, with the latter two again being similar (Additional file 1: Figure S1). However, the dominating species varied between land-use systems with *Salina* sp.01 ranking first in rainforest, *Salina* sp.02 in jungle rubber and oil palm plantations, but Lepidocyrtinae sp.04 in rubber plantations (Additional file 1: Table S1).

Of the 21 species found in only one of the four land-use systems, eleven species occurred exclusively in rainforest, seven in jungle rubber, two in rubber plantations and none in oil palm plantations (Additional file 1: Figure S2A). Rainforest and jungle rubber shared nine species, while three species only occur in two monoculture plantations. The number of species shared between the two landscapes (47 species) or the two seasons (45 species) was high (Additional file 1: Figure S2B, C). However, 14 species were found only in the Bukit Duabelas landscape and seven species were only found in the Harapan landscape. Further, 13 and 10 species were exclusively found in the rainy and dry season, respectively. All 21 species found across the four land-use systems were also found in both landscapes and both seasons, and mostly reached high abundances; they included 11 species of Entomobryidae, 7 of Paronellidae and 3 of Katiannidae (Additional file 1: Figure S2A). In contrast, species with a narrower distribution, i.e. not present in all of the land-use systems, generally reached low abundances (< 50 individuals in each land-use system) in both landscapes and seasons. Each land-use system except oil palm plantations also had several species found exclusively in that habitat. However, these species only reached low abundances or were found as single individuals only.

Species accumulation curves for all systems continuously increased, indicating that more species are to be expected with increasing sampling effort (Additional file 1: Figure S3). Contrary to the observed species richness, species accumulation curves suggest that the number of Collembola species in jungle rubber may exceed that in the rainforest. Similarly, the inverse Simpson index in jungle rubber (4.03 ± 1.24) exceeded that in rainforest (2.72 ± 1.24), with the latter being similar to that in oil palm (2.46 ± 2.18) and higher than that in rubber (1.87 ± 1.00; glm, F_3,60_ = 4.88, p = 0.005 for the effect of Land use); none of the interactions were significant (glm: F < 1.00 and p > 0.05).

### Community composition

**Fig. 3:**
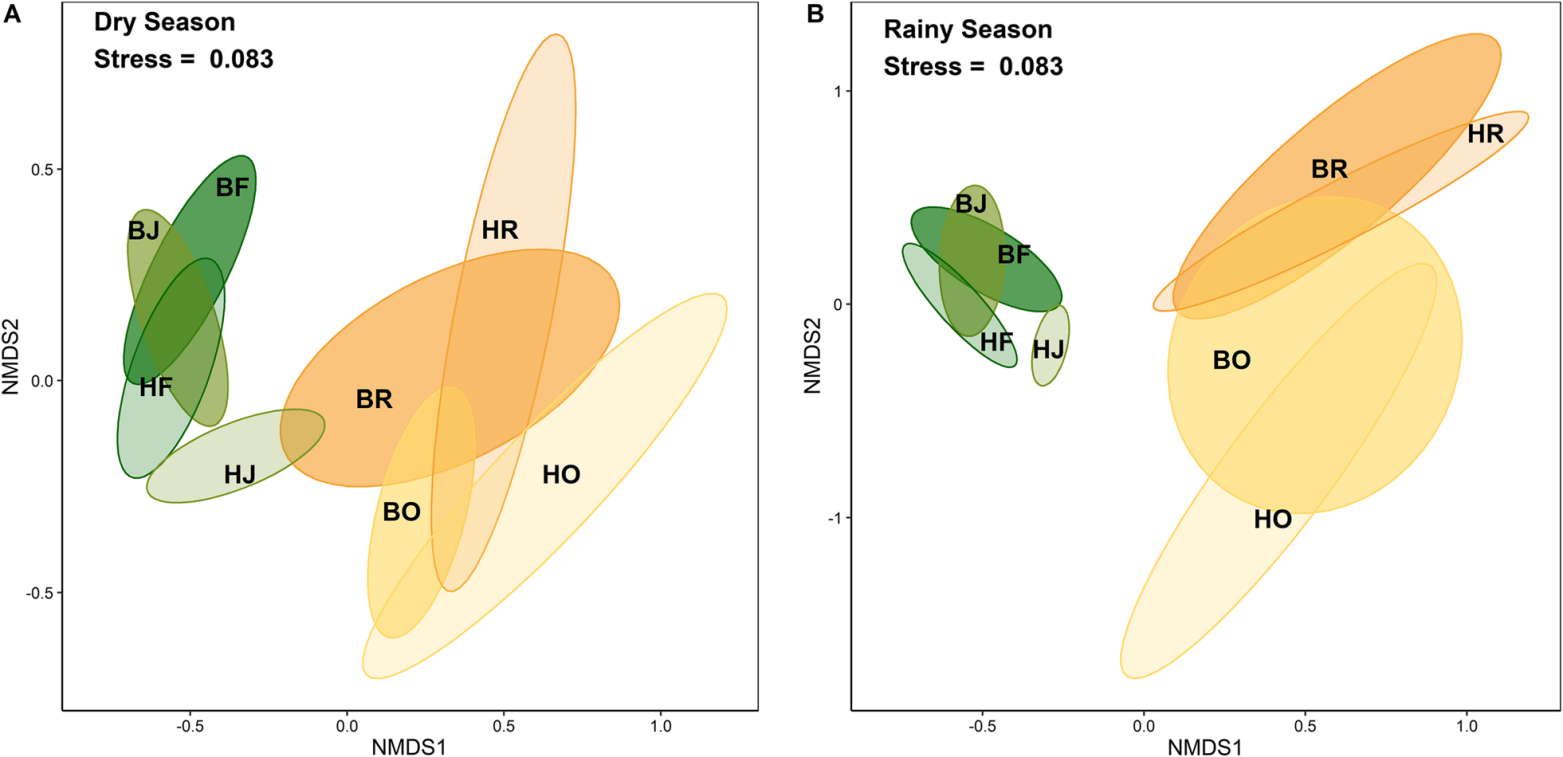
Non-metric multidimensional scaling (NMDS, k = 5) of arboreal Collembola communities in four land-use systems [rainforest (**F**), jungle rubber (**J**), rubber plantations (**R**) and oil palm plantations (**O**)] and two landscapes [Bukit Duabelas (**B),** Harapan (**H**)] in Jambi province, Sumatra, based on Bray–Curtis dissimilarities. Dry season (**A**) and rainy season (**B**); colored ellipses represent the 95% confidence ranges in the respective land-use system

Land use (Wilk’s lambda = 0.005, F_3,48_ = 30.53, p < 0.001), Landscape (Wilk’s lambda = 0.28, F_1,48_ = 15.30, p < 0.001) and Season (Wilk’s lambda = 0.60, F_1,48_ = 3.99, p = 0.002) significantly affected Collembola community composition, with the effect of Land use varying significantly with Landscape (Wilk’s lambda = 0.21, F_3,48_ = 4.01, p < 0.001), but not with Season (Wilk’s lambda = 0.59, F_1,48_ = 1.13, p = 0.32). The three-factor interaction was not significant (Wilk’s lambda = 0.49, F_3,48_ = 1.61, p = 0.06). NMDS separated rainforest and jungle rubber from the two monoculture plantation systems, more strongly in the rainy than in the dry season (Fig. 3). Confidence ranges in rainforest and jungle rubber were generally smaller and these two land-use systems clustered more closely than the monoculture plantation systems. Reflecting the significant interaction between Land use and Landscape, confidence ranges of rainforest and jungle rubber closely overlapped in the Bukit Duabelas landscape, but not in the Harapan landscape during both the dry and rainy season. Further, rubber and oil palm plantations overlapped in both the Bukit Duabelas and Harapan landscape during the dry season, while in the rainy season, rubber and oil palm plantations were separated in the Harapan but not in the Bukit Duabelas landscape.

**Fig. 4:**
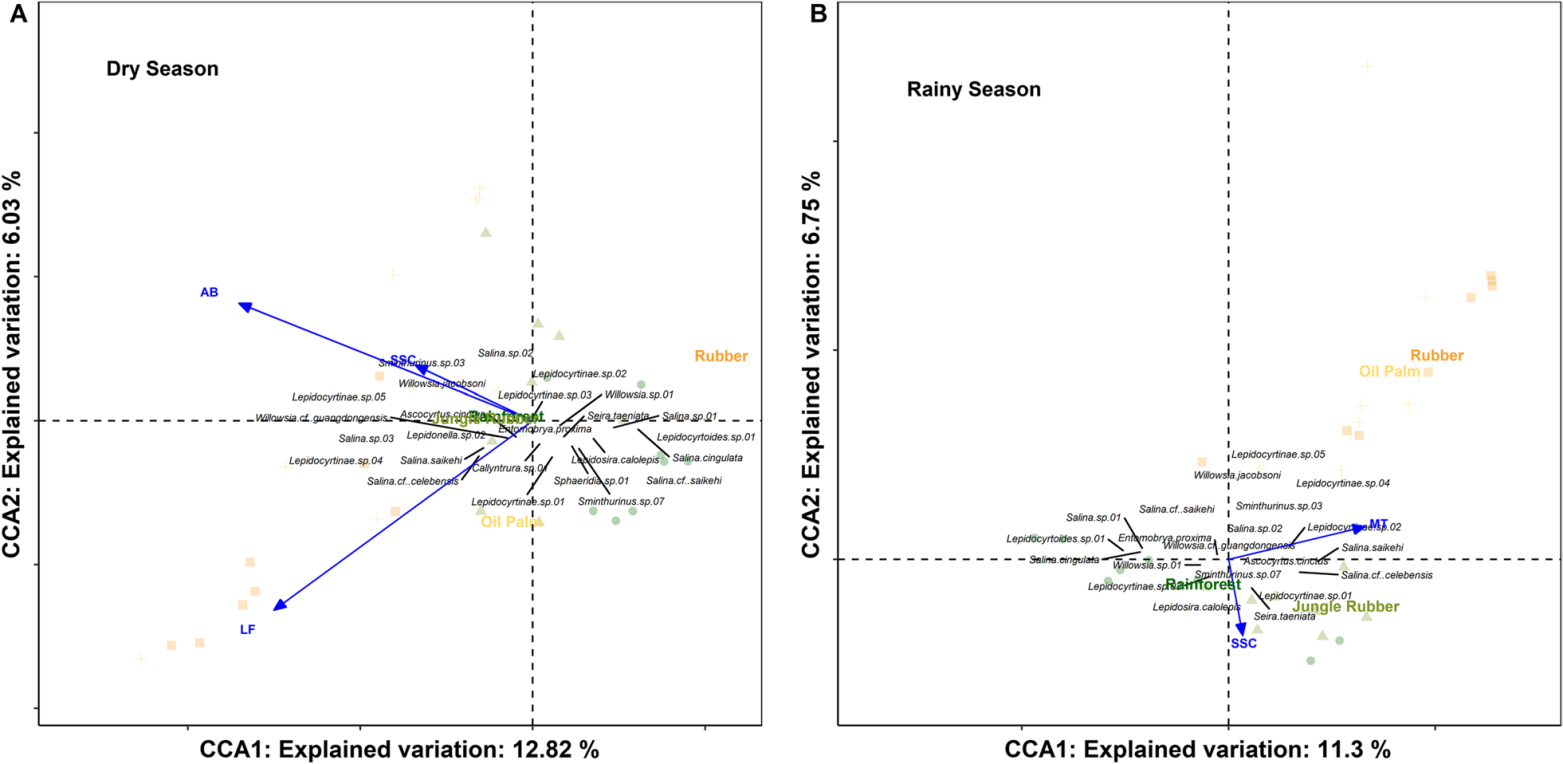
Canonical correspondence analysis (CCA) of arboreal Collembola species in four land-use systems (rainforest, jungle rubber, rubber plantation, and oil palm plantation) in the dry (**A**) and rainy season (**B**) as associated with environmental factors [aboveground biomass (**AGB**), litterfall (**LF**), mean temperature (**MT**), stand structural complexity (**SSC**)]. The length of arrows reflects the strength of the correlation between environmental variables and CCA axes

Forward selection of environmental variables in the CCA indicated that the environmental variables affecting Collembola community composition in the four land-use systems varied between seasons (Fig. 4). In the dry season, CCA axis 1 explained 12.86% and axis 2 explained 6.03% of the variation in species data. Three of the 12 environmental variables correlated significantly with Collembola community composition: aboveground biomass (F = 4.85, p = 0.002, R^2^ = 0.11), litterfall (F = 3.64, p = 0.002, R^2^ = 0.18) and stand structural complexity (F = 2.95, p = 0.002, R^2^ = 0.23). High aboveground biomass and stand structure complexity were associated with Collembola communities in rainforest, while litterfall was associated with jungle rubber. In rainforest *Salina* cf. *saikehi*, *Sphaeridia* sp.01, *Lepidosira calolepis*, *Lepidocyrtoides* sp.01 and *Sa. cingulata* were associated with high aboveground biomass; in jungle rubber Lepidocyrtinae sp.02 and *Salina* sp.02 were associated with high litterfall. Oil palm and rubber plantations were characterized by only few species. Oil palm was associated with low aboveground biomass and low stand structural complexity but high density of *Sminthurinus* sp.03 and *Willowsia jacobsoni.* Rubber was associated with low litterfall but high density of Lepidocyrtinae sp.04 and *Salina* sp.03.

In the rainy season, the CCA explained a similar fraction of the variation in species data as in the dry season, i.e. canonical axis 1 explained 11.27% and canonical axis 2 6.78%. Similar to the NMDS, CCA separated the four land-use systems, but compared to the dry season, the communities in oil palm and rubber were more similar. Only two factors correlated significantly with the community composition of Collembola, mean temperature (F = 4.60, p = 0.002, R^2^ = 0.11) and stand structural complexity (F = 3.68, p = 0.002, R^2^ = 0.18). Similar to the dry season, few species, such as Lepidocyrtinae sp.04 and Lepidocyrtinae sp.03, were associated with rubber and oil palm plantations and, in turn, with low stand structural complexity and high mean temperature. Species associated with high stand structural complexity in rainforest and jungle rubber included *Le. calolepis* and *Se taeniata*. Notably, in both seasons stand structural complexity significantly affected the community composition of Collembola contributing to the separation of rainforest and jungle rubber on one side and rubber and oil palm plantations on the other. Together with aboveground biomass in the dry season, it also contributed to the separation between rainforest and jungle rubber.

## Discussion

Conversion of rainforest into rubber and oil palm plantations is associated with a strong decrease in the abundance, species richness and diversity of arboreal Collembola. The differences were most pronounced between the more natural ecosystems, rainforest and jungle rubber, and the monoculture plantations of rubber and oil palm. The results support our first hypothesis suggesting that rainforest Collembola species suffer strongly from land-use conversion into monoculture plantations. Further, supporting our second hypothesis, Collembola were much less affected by the conversion into the extensive agroforestry system jungle rubber, than into monoculture plantations of rubber and oil palm. In fact, the abundance, species richness, species diversity and species composition of Collembola were similar in jungle rubber and rainforest. Supporting our third hypothesis, abundance and community composition of Collembola varied between seasons with this effect being most pronounced in monoculture plantations of rubber and oil palm.

### Collembola community composition

Two families, Paronellidae and Entomobryidae, dominated the Collembola communities in each of the four land-use systems. Paronellidae is a highly diverse family of Collembola in tropical rainforests, especially in Asia [50, 57]. Unlike most other Collembola, species of the family Paronellidae preferentially colonize aboveground habitats, such as canopies, epiphytes, trees, shrubs, grasses and occasionally leaf litter [58]. Among Paronellidae, species of the genus *Salina*, which only occurs in the tropics, dominated in both abundance and number of species [58]. *Salina* species are typically medium-sized Collembola with long appendages [59]. Of the 78 described species, three were present at our study sites (*Salina celebensis*, *Salina saikehi* and *Salina cingulata*) [59, 60], but we also found four species that are presumably new to science (*Salina* sp.01, *Salina* sp.02, *Salina* sp.03, *Salina* sp.04); further, *Salina* cf. *celebensis* and *Sa.* cf. *saikehi* may also be new to science. A similar dominance of Paronellidae among canopy Collembola has been reported from New Caledonia [61].

In contrast to Paronellidae, Entomobryidae is a cosmopolitan family and occurs in forests and other habitats across the world. Among Entomobryidae, five morphospecies from the subfamily Lepidocyrtinae comprised 65% of total Entomobryidae. Our results to some extent resemble the community composition of Collembola in the canopy of an Australian tropical rainforest, in which Entomobryidae dominated, but Paronellidae contributed only 7% to total Collembola abundance [62]. In general, only a few studies have investigated arboreal Collembola communities, limited to tropical and temperate forests or plantations [49, 62–65]. Although limited, these studies suggest that forest canopies provide ample habitat for Collembola, with Collembola community composition varying between regions, seasons and land-use systems.

Notably, arboreal Collembola communities at our study sites virtually did not overlap with those in litter and soil [55], indicating that Collembola communities in the canopies of tropical forests are distinct from those in soil. This supports earlier conclusions based on studies in subtropical rainforests in Australia [36, 37] and tropical rainforests in Africa [66] that Collembola communities in canopy habitats, such as suspended soil, suspended leaf litter, bark and epiphytes, are distinct from those of the forest floor. Similarly, Collembola communities on bark and in moss in the canopy of temperate forests have been found to be distinct from those in soil and moss on the forest floor [63], and in Japanese cedar plantations, Collembola community composition differed between canopy and forest floor [65]. Distinct communities in the canopy of trees and the forest floor have also been found for ants in cloud forests in Costa Rica [67], Lepidoptera in Bornean rainforest [27], bees, wasps and parasitoids in temperate forests [68], and microarthropods such as oribatid mites in Canadian temperate rainforest [69], and mesostigmata mites in Australian tropical rainforest [44]. Presumably, these differences reflect the necessity to adapt to the very different microclimatic conditions in the canopy of trees and on the forest floor. As a note of caution, however, canopy fogging may not effectively sample the fauna of canopy habitats such as epiphytes and suspended soil, as stressed by [70], and the fauna in these habitats may more closely resemble soil animal communities than suggested by our and other data based on canopy fogging.

Eleven species, four Paronellidae and seven Entomobryidae, made up 80% of the arboreal Collembola. Some of these species were found in each of the four land-use systems, typically reaching high abundances. However, the dominance ranks of these species varied among the different land-use systems, indicating different sensitivity to environmental factors. Generally, dominant species were similar in rainforest and jungle rubber and differed from those in rubber and oil palm plantations. This suggests that environmental factors and microhabitats in jungle rubber resemble those in rainforest, as confirmed by both NMDS and CCA. However, individual species also responded to the conversion of rainforest into jungle rubber as exemplified by *Salina* sp.01, which was the most dominant species in rainforest, but ranked at position six in jungle rubber similar to that in rubber (rank 4) and oil palm plantations (rank 6). In contrast, *Salina* sp. 02 was the sixth most abundant species in the rainforest, but ranked first in jungle rubber and oil palm plantations and second in rubber plantations. Presumably, *Salina* sp.02 is more tolerant to environmental conditions associated with open canopies, such as higher temperature and lower humidity, than *Salina* sp. 01. Some species, such as Lepidocyrtinae sp.03, *Se. taeniata* and *Willowsia* cf. *guangdongensis*, reached higher abundance in jungle rubber than in rainforest, supporting our conclusion that at species level Collembola also sensitively respond to the conversion of rainforest into jungle rubber. The dominance of *Salina* sp.02 and Lepidocyrtinae sp.04 in rubber and oil palm plantations suggests that these species are among the most tolerant Collembola species against harsher environmental conditions in monoculture plantations associated with low stand structural complexity and high temperature compared to rainforest and jungle rubber.

### Differences between land-use systems

The close similarity of Collembola density, species richness and community composition in jungle rubber and rainforest irrespective of season and landscape was somewhat surprising. We hypothesized that Collembola communities will be less affected by the conversion of rainforest into jungle rubber than into monoculture plantations of rubber and oil palm, but we did not expect Collembola communities in rainforest and jungle rubber to closely resemble each other. The results suggest that, contrasting herbivores, canopy Collembola communities rely on factors other than plant (tree) species diversity. Although jungle rubber contains native tree species, tree diversity is strongly reduced compared to rainforest [52]. By contrast, canopy structure and associated microclimatic conditions (cooler and more humid) are similar in rainforest and jungle rubber, suggesting that dense canopy and associated microhabitat conditions are the most important factors driving Collembola communities and this is supported by our NMDS and CCA results. As a note of caution, the results of our study might be biased by our sampling technique. We sampled Collembola by fogging target canopies and thereby we likely overestimated the density of Collembola in plantations due to more open canopies, in particular in monoculture plantation systems. Differences between rainforest and jungle rubber on one side and monoculture plantations on the other, are therefore likely to be underestimated.

Both NMDS and CCA clearly separated jungle rubber and rainforest from rubber and oil palm plantations. In jungle rubber and rainforest this separation was independent of season, whereas the communities in rubber and oil palm plantations differed most in the dry season. The more pronounced differences during the dry season are likely associated with rubber trees shedding their leaves [71] as well as low abundance and diversity of epiphytes [8]. In Venezuelan cloud forests, Collembola have been reported to be the most abundant group of microarthropods inhabiting epiphytic bromeliads aside from Acari, suggesting that epiphytes form an important habitat for arboreal Collembola [39]. Considering the similarity of Collembola communities in jungle rubber and rainforest as well as that of other arboreal arthropods, such as ants [22], spiders [25] and parasitoid wasps [56], jungle rubber may represent an ideal land-use system for arboreal arthropods from a conservation but also a functional perspective. However, from a socioeconomic perspective, jungle rubber is no longer attractive because it requires high labor intensity but yields only low income [11, 72].

Generally, the strong differences in Collembola communities between rainforest and jungle rubber on one side, and rubber and oil palm plantations on the other, resemble soil Collembola communities, which also have been shown to differ strongly in abundance and community composition between forests and agricultural replacement systems [55, 73]. These results are also consistent with studies on other arthropod taxa in arboreal [22, 74] and forest floor habitats [75] at our study sites.

Collembola communities are structured by both environmental factors and the availability of food resources. As Collembola feed predominantly on detritus and associated microorganisms, Collembola community composition has been shown to be rather independent of the diversity of plants [76] and forest type/tree species [77]. Rather than reduced plant diversity and tree identity, changes in microclimate and other environmental factors associated with the conversion of rainforest into plantation systems are likely responsible for the detrimental effects on Collembola communities in both the canopy and soil of plantations. Plantation environments thus select for specific communities more resistant to harsher environmental conditions. Conversion of less-profitable jungle rubber into more profitable systems such as oil palm plantations, as frequently occurring in our study region, compounds the detrimental effects of rainforest conversion on Collembola communities in the canopy and soil.

### Changes with season

Changes in Collembola communities with season were much less pronounced than those between land-use systems. Notably, neither the abundance nor the diversity of Collembola significantly varied with season and as indicated by the lack of significant interactions between season and land-use system, this was consistent across the studied land-use systems. The same pattern was found in Braconid wasps [56]. This was unexpected since season has been reported to more strongly affect arthropod communities than tree species [78]. Because most Collembola are soft-bodied arthropods sensitive to desiccation, we assumed they would suffer during the dry season, particularly in monoculture plantations with more open canopies. Indeed, canopy Collembola in wet and dry tropical forests, as well as temperate forests in Mexico, showed significant seasonal variation in abundance and species composition [49]. Generally, most species in the tropics reach higher abundance in the rainy season than in the dry season, but presumably the dry season at our sites is not strong enough to limit the activity and density of arboreal Collembola. In fact, even during the dry season, there are regular rainfall events in lowland Sumatra, especially in Jambi Province [71, 79].

As indicated by CCA, differences in arboreal Collembola communities between the dry and rainy seasons were mainly due to differences in stand structural complexity, aboveground biomass, litterfall and temperature in the four land-use systems. In part, this was unexpected, as we assumed canopy openness and tree species would be among the most important factors affecting the structure of arboreal Collembola communities. This assumption was based on results of previous studies showing vegetation type to significantly affect Collembola community composition [49]. However, the CCA identified stand structural complexity as the most important factor structuring Collembola communities in both seasons. Stand structural complexity is calculated from a number of stand characteristics including stem density, mean diameter at breast height, basal area and canopy cover [80]. For Collembola communities in litter and soil, plant richness and plant biomass have been shown to significantly affect the abundance of epedaphic Collembola in secondary forests [81], which presumably reflects their dependence on the structural complexity of the habitat rather than plant richness per se. In our study, plant species richness did not significantly affect Collembola abundance, although it may also contribute to stand structural complexity. Land-use systems with more complex stand structures increase the vertical stratification and crown plasticity, which leads to denser canopies [82] providing more suitable niches for arboreal microarthropods such as Collembola.

In addition to stand structural complexity, Collembola communities in the dry season were structured by aboveground biomass and litterfall, and in the rainy season by temperature, reflecting the difference between the two more natural systems and the two monoculture plantations. Aboveground plant biomass is high in rainforests, which relates to tree height and basal area as well as age [83]. Tropical rainforests typically include tall mature trees and dense canopies colonized by diverse epiphyte species, which provide favorable habitats for Collembola. Aboveground plant biomass also has been shown to represent an important environmental predictor for jumping spiders (Salticidae) at our study sites [25] and Collembola may form an important part of their diet.

The third significant environmental predictor in the dry season, litterfall, was higher in rainforest than in the other land-use systems [80]. Litterfall is essential for the formation of soil organic matter. Presumably, high litterfall correlates with the amount of leaf debris accumulating in the canopy. Jungle rubber canopies are almost as dense as rainforest canopies and comprise a similar abundance and diversity of epiphytes [8], contributing to the accumulation of leaf debris in the canopy. Thus, suspended soil can be formed from the trapped litter combined with components from epiphytes. In the canopy of lowland dipterocarp rainforests in Borneo bird’s nest ferns (*Asplenium nidus* complex), which capture large amounts of litter, have been shown to favor diverse invertebrate communities including Collembola [84].

The second significant environmental predictor in the rainy season was temperature. Forest and jungle rubber had similar microclimatic conditions (air temperature and humidity), which differed from those in monoculture plantations by being colder and more humid [8, 85]. As stressed above, Collembola benefit from humid conditions and, although conditions are generally getting colder and more humid in the rainy season, Collembola still benefit from colder and more humid conditions in rainforest and jungle rubber.

## Conclusion

Most arthropods in Indonesian forest canopies are still unexplored and this is especially true for small microarthropod taxa such as Collembola. Unlike more well-studied arboreal groups, such as ants, butterflies and spiders living as herbivores and predators, Collembola represent the poorly understood detritivore component of the canopy food web. Our study is the first to investigate in detail the effect of land-use change on arboreal Collembola communities, which form an important component of the canopy food web in tropical rainforests and arboreal agricultural replacement systems.

Collembola abundance, diversity and community composition strongly responded to changes in land use but varied little with season. The differences were most pronounced between rainforest and jungle rubber on one side, and monoculture plantations of rubber and oil palm on the other. The close similarity of Collembola communities in rainforest and jungle rubber suggest that rubber (and other) agroforestry systems provide biodiversity-friendly alternatives to monoculture plantations. Of the 68 (morpho)species found, certain species reached high abundances in each of the land-use systems, but others sensitively responded to land-use change. Each land-use system except oil palm plantations also had exclusive species. Stand structural complexity was identified as an important factor shaping arboreal Collembola community composition in both the dry and wet season. In the dry season, aboveground plant biomass and litterfall correlated with Collembola community composition, while in the rainy season the second most significant environmental predictor was temperature. Overall, the results provide the first insight into arboreal Collembola communities in tropical rainforests and arboreal agricultural replacement systems, one of the most abundant but least explored arthropod taxa in the canopies of tropical forests. As detritivore microarthropods, they add to the complexity of the canopy food web and contribute to the diet of predators, thus affecting interactions between herbivore insects as potential pest species in plantations and predator arthropods.

## Materials and methods

### Study sites

The study was conducted in the lowlands of Jambi province, Sumatra, Indonesia as part of the EFForTS project (Ecological and socioeconomic functions of tropical lowland rainforest transformation systems) [85] (Additional file 1: Figure S4). The region is characterized by a tropical climate with a period of lower precipitation from May to August (dry season) and a rainy season from November to March [85].

Collembola community composition was studied in 50 m × 50 m plots in the rainforest and three agricultural replacement systems, including jungle rubber agroforest and monoculture plantations of rubber (*Hevea brasiliensis*) and oil palm (*Elaeis guineensis*) (Additional file 1: Figure S5). Replicated experimental plots were established for each land-use system in each of two landscape areas, near Bukit Duabelas National Park and Harapan Rainforest (see [85]). In total, we sampled arboreal Collembola from 32 plots in each the dry and rainy season.

The lowland rainforest sites (henceforth referred to as rainforest) represent’ primary degraded forest’ that served as reference [6, 52]. Jungle rubber agroforest is an extensive rubber plantation system with minimum management, originating from the planting of rubber trees into rainforest and resulting in a patchy tree structure due to the natural regeneration of rainforest trees and continuous planting of rubber seedlings [52, 72, 86]. The studied rubber and oil palm plantations originated from rainforest or jungle rubber remnants that were converted into monoculture plantations and were owned by smallholders. At the time of sampling, plantation ages ranged between 7 and 16 years for both rubber and oil palm [52, 80, 85].

### Sampling

Arboreal Collembola were sampled from May to July 2013 in the dry season and November 2013 to March 2014 in the rainy season. Collembola were collected by canopy fogging, using the Swingfog^®^ SN50 fogger (Swingtec, Isny, Germany) to apply a mixture of 50 ml DECIS 25 EC^®^ (Bayer Crop Science, Jakarta, Indonesia; active ingredient deltamethrin, 25 g/L) dissolved in four liters of petroleum oil to three target canopies per plot. The target canopies were selected by visually identifying three locations with canopies of representative nature to the entire plot; i.e. canopy gaps and fallen trees were avoided. Underneath each target canopy, we placed 16 funnel traps of 1 m × 1 m suspended from ropes tied to height-adjustable tent poles, each fitted with 250 ml wide-neck PE flasks filled with 100 ml 96% ethanol. Fogging was conducted between 6 to 10 AM to minimize the influence of wind, and arthropods were collected 2 h after the insecticide application, transferred into 50 ml tubes with 96% ethanol and then stored at − 20 °C. All arthropod specimens were counted and sorted to order level under a stereomicroscope.

### Morphological examination

Collembola specimens were determined based on morphological characters and grouped into morphospecies using a Zeiss Stemi 508 microscope with appropriate magnification. Two specimens were retained from each morphospecies as wet vouchers and 2–3 specimens were selected to be slide-mounted. The specimens were cleared using Nesbitt’s solution and then mounted using Hoyer's solution [87]. For determining Collembola to species level, we used a compound microscope (Zeiss, Axio Lab.A1) at suitable magnification. The checklist and keys to Indonesian Collembola [88] were used for determining Collembola beyond family level. Morphospecies within genera were identified using original descriptions of species from publications on Southeast Asian Collembola [89–98]. Damaged specimens were identified to the highest taxonomic level possible and included for calculating the abundance of the respective level.

### Statistical analysis

Statistical analyses were carried out using R v 4.1.2 [99] with R Studio interface [100] and visualized with *ggplot2* [101]. Each data set, abundance (ind./m^2^; calculated from the sum of three replicate canopies per plot divided by 48, i.e. the total number of traps), species richness (number of species per plot) and community composition, was explored to identify the most appropriate model for the data distribution for analysis. The initial model for each data set included the factors Land use (rainforest, jungle rubber, rubber plantations, and oil palm plantations), Landscape (Bukit Duabelas and Harapan) and Season (dry and rainy season), and the interactions between all factors as fixed effects. Abundance and beta-diversity were analyzed using generalized linear models (glm) with family Gamma and log link function (stats::glm [99]), and species richness (number of species per plot) using linear models (lm, stats::lm [99]). Models were simplified in a stepwise way by removing non-significant terms to arrive at the final model. Post-hoc analysis was carried out using Tukey’s HSD test at α = 0.05 (multcomp::glht [102]). Rank abundance curves between the land-use systems were calculated (vegan::radfit [103]) and visualized using the RankAbund package [104].

The effects of rainforest conversion on arboreal Collembola communities were visualized separately for each season using non-metric multidimensional scaling (NMDS; vegan::mds, Bray Curtis dissimilarity index; k = 5, stress = 0.083 for both seasons). Multivariate analysis of variance (MANOVA) was used to analyze the effect of Land use, Landscape and Season, and their interactions on Collembola community composition. The length of the gradient was > 3.5 and therefore canonical correspondence analysis (CCA, vegan::cca and ade4, [105–109]) was used to assess the relationship between species composition and environmental variables. Only species that were present in at least five plots were included in the analyses.

We explored the effect of 12 environmental variables on community composition of arboreal Collembola. Mean annual temperature (%) and humidity (%) in 2013 were derived from hourly measurements with thermohygrometers (Galltec Mela^®^) placed at 2 m height, and stored using a UIT Log-Trtans 16-GPRS data logger [85, 110]. Canopy openness was derived from hemispherical photographs taken at 1.2 m above the ground from 32 positions within each core plot in 2013 (Canon EOS 700D SLR camera and SIGMA 4.5 mm F2.8 EX DC circular fisheye lens), followed by applying the ‘minimum thresholding algorithm’ using the ‘ImageJ’ software [80, 85]. Canopy structure was assessed as the Stand Structural Complexity Index (SSCI), and the Effective Number of Layers (ENL), both calculated from data obtained with a FARO Focus terrestrial laser scanner at the center of each plot in September and October 2016 (methodological details in [111, 112], unpublished data from C. Zemp & H. Kreft).

Management intensity in each plot was quantified as the Land Use Intensity Index (LUI), which contains data on both organic and anorganic fertilization, mechanical weeding or herbicide use, liming (CaCO_3_) and planting density [113]. Leaf litterfall was measured monthly between March 2013 and April 2014 by weighing dried leaves from 16 litter traps in each plot of forest, jungle rubber and rubber plantation, and two fully grown oil palm fronds in each oil palm plantation plot where no natural litterfall occurs [80, 85]. Aboveground tree biomass (AGB) was calculated by applying land-use specific allometric equations to measurements of height and diameter of all trees in each plot with a diameter at breast height (dbh) ≥ 10 cm, as well as trees with a dbh between 2 and 10 cm in two subplots per plot, taken between August and September 2012 (details in [80, 85]). And finally, we used tree species richness and abundance of all trees with dbh ≥ 10 cm in each plot, and plant species richness and abundance of all vascular plants (including trees) in five subplots nested within each plot (for detailed plot design information see [85]; for methodological information and data on plant surveys see [52]; partially unpublished data K. Rembold & H. Kreft).

We used the adjusted coefficient of multiple determination (R^2^_a_) and forward selection (vegan::ordi2step, permutation = 999) to identify the environmental variables significantly correlated with Collembola community composition [114]. The significance of the constrained ordination was tested by permutation tests.

## Supplementary Information

**Additional file 1: Figure S1. **Species rank-abundance curves of arboreal Collembola in the four land-use systems studied (rainforest, jungle rubber, rubber plantation, oil palm plantation).** Table S1.** Relative abundance and rank of Collembola species in the four land-use systems (rainforest, jungle rubber, rubber plantation, oil palm plantation) in Jambi Province, Sumatra, Indonesia.** Figure S2.** Venn diagrams of shared and exclusive Collembola species in the four land-use systems (rainforest, jungle rubber, rubber plantation, oil palm plantation) (A), two landscapes (Bukit Duabelas, Harapan) (B) and two seasons (dry, rainy) (C).** Figure S3.** Species accumulation curves showing accumulation rates of new species in the studied four land-use systems (rainforest, jungle rubber, rubber plantation, oil palm plantation) in Jambi Province, Sumatra, Indonesia.** Figure S4.** Location of the 32 study plots in two landscapes near Bukit Duabelas National Park and Harapan Rainforest in Jambi Province, Sumatra, Indonesia (from [24]).** Figure S5.** Overview of the investigated land-use systems: Lowland rainforest (A), jungle rubber (B), rubber plantation (C) and oil palm plantation (D). Photos by Jochen Drescher.

## Data Availability

The data that support the findings of this study are openly available on the GRO Göttingen Research Online data repository at https://data.goettingen-research-online.de/ under the following DOIs: https://doi.org/10.25625/K9PBWM (species-abundance per plot) and https://doi.org/10.25625/OZL8NN (environmental variables per plot).
