# Supplementary material for: Response of arboreal Collembola communities to the conversion of lowland rainforest into rubber and oil palm plantations
Source: BMC Ecol Evol. 2022 Dec 14;22:144. doi: 10.1186/s12862-022-02095-6 (PMC9753237; doi:10.1186/s12862-022-02095-6)

# **ADDITIONAL FILES**

#### Figure S1. Species rank-abundance curves of arboreal Collembola in the four land-use systems studied (rainforest, jungle rubber, rubber plantation, oil palm plantation).


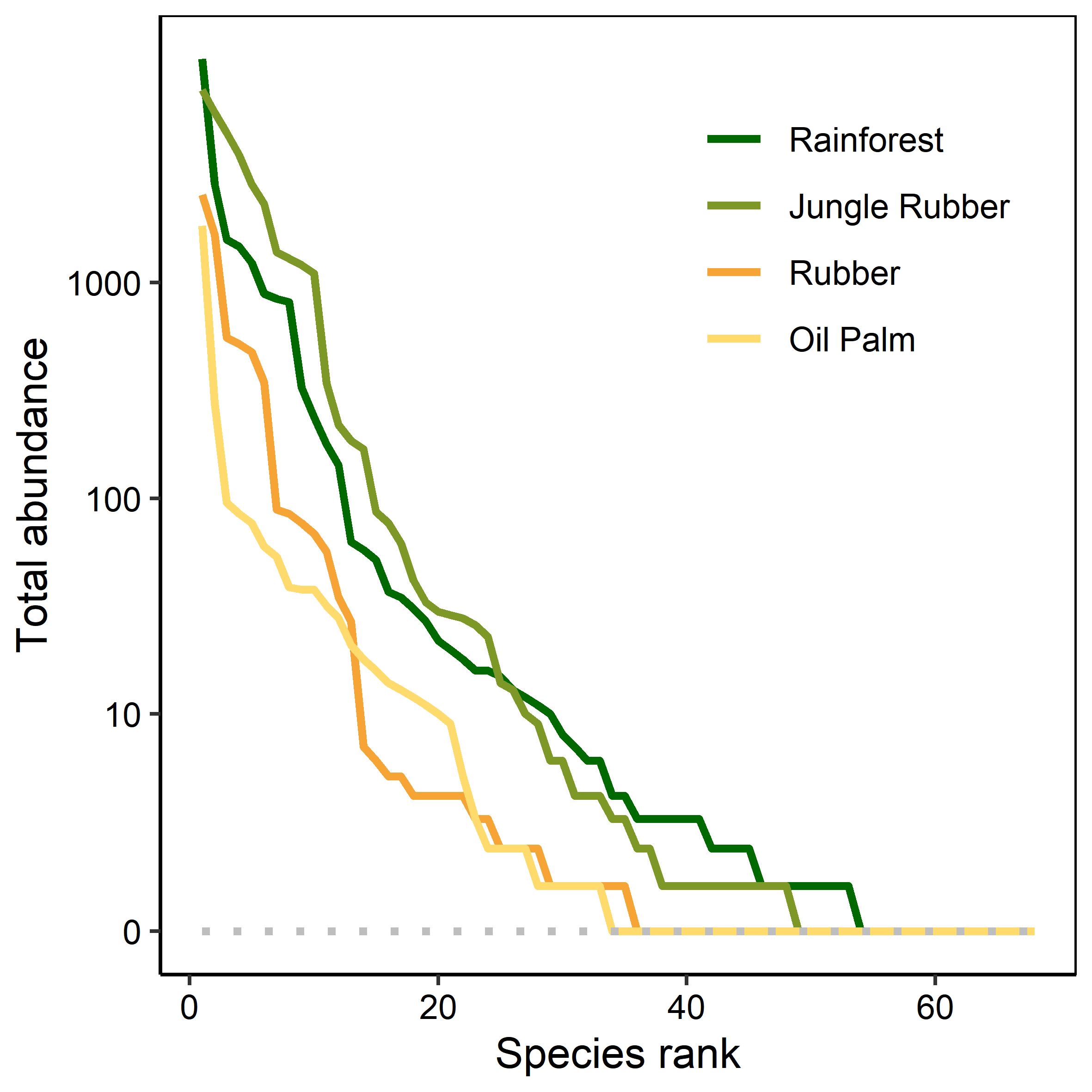


#### Table S1. Relative abundance and rank of Collembola species in the four land-use systems (rainforest, jungle rubber, rubber plantation, oil palm plantation) in Jambi Province, Sumatra, Indonesia.

| Nr. | Morpho(species) | Rainforest | | Jungle rubber | | Rubber | | Oil palm | |
| --- | --- | --- | --- | --- | --- | --- | --- | --- | --- |
|  |  | Mean ± SD | Rank | Mean ± SD | Rank | Mean ± SD | Rank | Mean ± SD | Rank |
| 1 | *Ascocyrtus cinctus* | 0.001 ± 0.001 | 25 | 0.002 ± 0.005 | 18 | 0.001 ± 0.002 | 17 | 0.007 ± 0.016 | 13 |
| 2 | *Callyntrura kudatensis* | 0 | 55 | 0 | 67 | 0.003 ± 0.009 | 19 | 0.005 ± 0.014 | 17 |
| 3 | *Callyntrura* sp.01 | 0.002 ± 0.007 | 30 | 0.001 ± 0.001 | 24 | 0.003 ± 0.010 | 23 | 0 | 58 |
| 4 | *Callyntrura* sp.02 | < 0.001 | 51 | < 0.001 | 34 | 0 | 45 | 0 | 42 |
| 5 | *Callyntrura* sp.03 | 0 | 63 | 0 | 50 | 0.007 ± 0.016 | 14 | 0.003 ± 0.008 | 19 |
| 6 | *Callyntrura* sp.04 | < 0.001 | 47 | < 0.001 | 35 | 0 | 62 | 0.001 ± 0.005 | 33 |
| 7 | *Callyntrura* sp.05 | 0.002 ± 0.004 | 23 | < 0.001 | 45 | 0 | 49 | 0 | 47 |
| 8 | *Callyntrura* sp.06 | 0.000 ± 0.001 | 36 | 0 | 57 | 0 | 60 | 0 | 48 |
| 9 | *Callyntrura* sp.07 | 0 | 68 | 0 | 61 | 0.002 ± 0.007 | 21 | 0 | 45 |
| 10 | *Callyntrura* sp.08 | < 0.001 | 39 | < 0.001 | 40 | 0 | 53 | 0 | 60 |
| 11 | *Callyntrura* sp.09 | 0 | 67 | < 0.001 | 46 | 0 | 59 | 0 | 40 |
| 12 | *Callyntrura* sp.10 | 0 | 60 | < 0.001 | 39 | 0 | 43 | 0 | 56 |
| 13 | *Callyntrura* sp.11 | < 0.001 | 46 | 0 | 51 | < 0.001 | 30 | 0 | 38 |
| 14 | *Calvatomina* sp.01 | 0.002 ± 0.004 | 16 | 0 | 64 | 0 | 66 | 0 | 35 |
| 15 | *Calvatomina* sp.02 | 0.001 ± 0.003 | 26 | 0 | 55 | 0 | 61 | 0 | 44 |
| 16 | *Calvatomina* sp.03 | < 0.001 | 38 | 0 | 49 | 0 | 63 | 0 | 61 |
| 17 | *Calvatomina* sp.04 | 0 | 62 | < 0.001 | 41 | 0 | 44 | 0 | 46 |
| 18 | *Calvatomina* sp.05 | < 0.001 | 48 | 0 | 53 | 0 | 57 | 0 | 55 |
| 19 | *Calvatomina* sp.06 | 0 | 57 | 0 | 66 | 0.000 ± 0.001 | 34 | 0 | 50 |
| 20 | *Dicranocentroides* sp.01 | 0 | 66 | < 0.001 | 47 | 0 | 65 | 0 | 52 |
| 21 | *Entomobrya* cf. *proxima* | 0.003 ± 0.003 | 17 | < 0.001 | 44 | 0 | 56 | 0 | 54 |
| 22 | *Entomobrya proxima* | 0.003 ± 0.003 | 14 | 0.002 ± 0.003 | 15 | < 0.001 | 24 | 0.016 ± 0.044 | 21 |
| 23 | *Hypogastrura* sp.01 | < 0.001 | 44 | 0.017 ± 0.059 | 12 | 0 | 40 | 0.001 ± 0.003 | 30 |
| 24 | *Hypogastrura* sp.02 | 0 | 61 | 0.000 ± 0.001 | 29 | 0 | 50 | 0 | 41 |
| 25 | *Hypogastrura* sp.03 | 0 | 54 | 0.001 ± 0.003 | 26 | 0.000 ± 0.001 | 28 | 0 | 66 |
| 26 | Lepidocyrtinae sp.01 | 0.208 ± 0.240 | 2 | 0.161 ± 0.207 | 2 | 0.000 ± 0.001 | 18 | 0.013 ± 0.043 | 18 |
| 27 | Lepidocyrtinae sp.02 | 0.000 ± 0.002 | 29 | 0.038 ± 0.044 | 7 | 0.005 ± 0.014 | 9 | 0.017 ± 0.069 | 3 |
| 28 | Lepidocyrtinae sp.03 | 0.053 ± 0.073 | 4 | 0.158 ± 0.133 | 3 | 0.001 ± 0.003 | 15 | 0.007 ± 0.024 | 14 |
| 29 | Lepidocyrtinae sp.04 | 0.002 ± 0.005 | 13 | < 0.001 | 33 | 0.538 ± 0.308 | 1 | 0.164 ± 0.238 | 2 |
| 30 | Lepidocyrtinae sp.05 | 0.000 ± 0.001 | 40 | 0.000 ± 0.001 | 32 | 0.001 ± 0.002 | 16 | 0.116 ± 0.246 | 5 |
| 31 | Lepidocyrtoides sp.01 | 0.012 ± 0.011 | 10 | < 0.001 | 42 | 0 | 37 | 0 | 67 |
| 32 | *Lepidonella* sp.01 | 0.002 ± 0.004 | 21 | 0 | 62 | 0 | 52 | 0 | 51 |
| 33 | *Lepidonella* sp.02 | 0.000 ± 0.001 | 34 | < 0.001 | 38 | < 0.001 | 31 | 0.011 ± 0.038 | 20 |
| 34 | *Lepidonella* sp.03 | 0 | 59 | 0 | 60 | 0.000 ± 0.002 | 29 | 0.001 ± 0.005 | 31 |
| 35 | *Lepidosira calolepis* | 0.089 ± 0.064 | 3 | 0.057 ± 0.070 | 8 | 0.002 ± 0.007 | 22 | 0.001 ± 0.002 | 25 |
| 36 | *Salina celebensis* | < 0.001 | 52 | < 0.001 | 37 | 0.014 ± 0.055 | 8 | 00 | 65 |
| 37 | *Salina* cf. *celebensis* | 0.003 ± 0.008 | 19 | 0.056 ± 0.100 | 5 | 0.040 ± 0.081 | 6 | 0.018 ± 0.053 | 10 |
| 38 | *Salina* cf. *saikehi* | 0.014 ± 0.016 | 9 | 0.000 ± 0.001 | 25 | 0.005 ± 0.018 | 10 | 0.000 ± 0.001 | 24 |
| 39 | *Salina cingulata* | 0.027 ± 0.030 | 8 | 0.002 ± 0.004 | 21 | 0.004 ± 0.010 | 12 | 0.011 ± 0.041 | 9 |
| 40 | *Salina saikehi* | 0.001 ± 0.002 | 32 | 0.023 ± 0.035 | 10 | 0.008 ± 0.024 | 11 | 0.033 ± 0.062 | 4 |
| 41 | *Salina* sp.01 | 0.398 ± 0.257 | 1 | 0.092 ± 0.130 | 6 | 0.026 ± 0.086 | 4 | 0.022 ± 0.056 | 6 |
| 42 | *Salina* sp.02 | 0.077 ± 0.124 | 5 | 0.223 ± 0.202 | 1 | 0.159 ± 0.250 | 2 | 0.419 ± 0.401 | 1 |
| 43 | *Salina* sp.03 | 0 | 56 | 0.000 ± 0.001 | 28 | 0.024 ± 0.079 | 5 | 0.000 ± 0.001 | 26 |
| 44 | *Salina* sp.04 | 0.001 ± 0.003 | 18 | < 0.001 | 36 | 0 | 51 | 0 | 36 |
| *45* | *Seira taeniata* | 0.039 ± 0.031 | 6 | 0.053 ± 0.076 | 9 | 0.000 ± 0.001 | 20 | 0.002 ± 0.010 | 22 |
| 46 | *Sminthurinus* sp.01 | 0.000 ± 0.001 | 28 | 0 | 68 | 0 | 67 | 0 | 49 |
| 47 | *Sminthurinus* sp.02 | 0.001 ± 0.001 | 24 | 0 | 54 | 0 | 38 | 0 | 34 |
| 48 | *Sminthurinus* sp.03 | 0.001 ± 0.001 | 33 | 0.000 ± 0.001 | 20 | < 0.001 | 26 | 0.021 ± 0.035 | 7 |
| 49 | *Sminthurinus* sp.04 | < 0.001 | 49 | 0 | 59 | 0.002 ± 0.007 | 33 | 0.030 ± 0.115 | 8 |
| 50 | *Sminthurinus* sp.05 | < 0.001 | 50 | 0.000 ± 0.000 | 31 | 0.014 ± 0.023 | 7 | 0.014 ± 0.028 | 16 |
| 51 | *Sminthurinus* sp.06 | < 0.001 | 53 | 0.005 ± 0.009 | 14 | 0 | 39 | 0 | 57 |
| 52 | *Sminthurinus* sp.07 | 0.008 ± 0.011 | 12 | 0.012 ± 0.021 | 11 | 0 | 54 | 0.019 ± 0.062 | 15 |
| 53 | *Sminthurinus* sp.08 | < 0.001 | 41 | 0.002 ± 0.004 | 16 | < 0.001 | 32 | 0.027 ± 0.048 | 12 |
| 54 | *Sminthurinus* sp.09 | 0 | 58 | < 0.001 | 30 | 0 | 48 | 0.001 ± 0.005 | 29 |
| 55 | *Sphaeridia* sp.01 | 0.001 ± 0.001 | 22 | 0.001 ± 0.001 | 23 | 0 | 64 | 0.000 ± 0.001 | 28 |
| 56 | *Sphyrotheca* sp.01 | 0.002 ± 0.004 | 15 | 0.001 ± 0.003 | 27 | 0 | 46 | 0 | 68 |
| 57 | *Sphyrotheca* sp.02 | < 0.001 | 45 | 0.003 ± 0.009 | 17 | 0.000 ± 0.001 | 25 | 0 | 39 |
| 58 | *Sphyrotheca* sp.03 | 0.001 ± 0.002 | 31 | 0.001 ± 0.005 | 19 | 0 | 41 | 0 | 63 |
| 59 | *Sphyrotheca* sp.04 | 0.001 ± 0.002 | 20 | < 0.001 | 43 | 0.000 ± 0.002 | 35 | 0 | 59 |
| 60 | *Sphyrotheca* sp.05 | 0.000 ± 0.001 | 35 | 0 | 63 | 0 | 55 | 0 | 62 |
| 61 | *Sphyrotheca* sp.06 | < 0.001 | 42 | 0 | 52 | 0 | 68 | 0.000 ± 0.001 | 32 |
| 62 | *Sphyrotheca* sp.07 | 0 | 64 | < 0.001 | 48 | 0 | 47 | 0 | 37 |
| 63 | *Superodontella* sp.01 | < 0.001 | 43 | 0 | 65 | 0 | 36 | 0 | 64 |
| 64 | *Willowsia* cf. *guangdongensis* | 0.038 ± 0.031 | 7 | 0.079 ± 0.094 | 4 | 0.130 ± 0.190 | 3 | 0.002 ± 0.005 | 23 |
| 65 | *Willowsia* *hyalina* | 0 | 65 | 0.001 ± 0.004 | 22 | 0 | 58 | 0 | 53 |
| 66 | *Willowsia* *jacobsoni* | 0.001 ± 0.001 | 27 | 0 | 56 | 0.002 ± 0.006 | 27 | 0.014 ± 0.020 | 11 |
| 67 | *Willowsia* sp.01 | 0.007 ± 0.006 | 11 | 0.006 ± 0.007 | 13 | 0.009 ± 0.029 | 13 | 0.001 ± 0.005 | 27 |
| 68 | *Willowsia* sp.02 | 0.000 ± 0.000 | 37 | 0 | 58 | 0 | 42 | 0 | 43 |

#### Figure S2. Venn diagrams of shared and exclusive Collembola species in the four land-use systems (rainforest, jungle rubber, rubber plantation, oil palm plantation) (A), two landscapes (Bukit Duabelas, Harapan) (B) and two seasons (dry, rainy) (C).

**
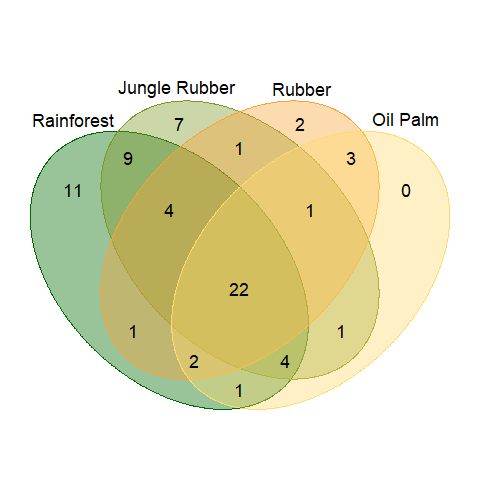

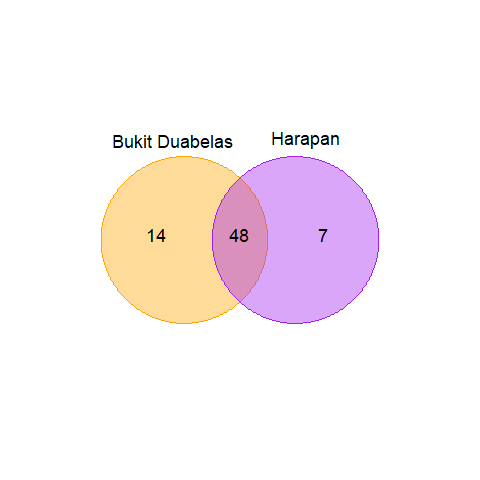
**

B

A


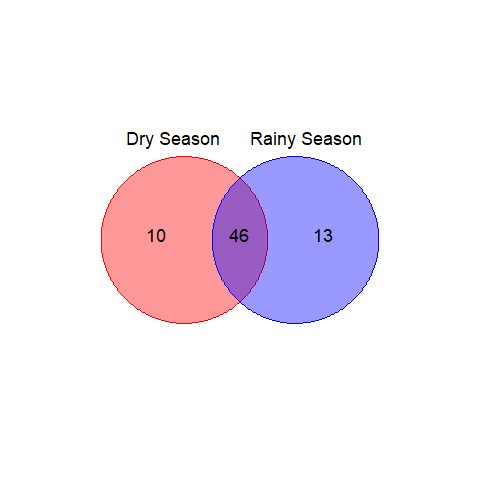


C

#### Figure S3. Species accumulation curves showing accumulation rates of new species in the studied four land-use systems (rainforest, jungle rubber, rubber plantation, oil palm plantation) in Jambi Province, Sumatra, Indonesia.


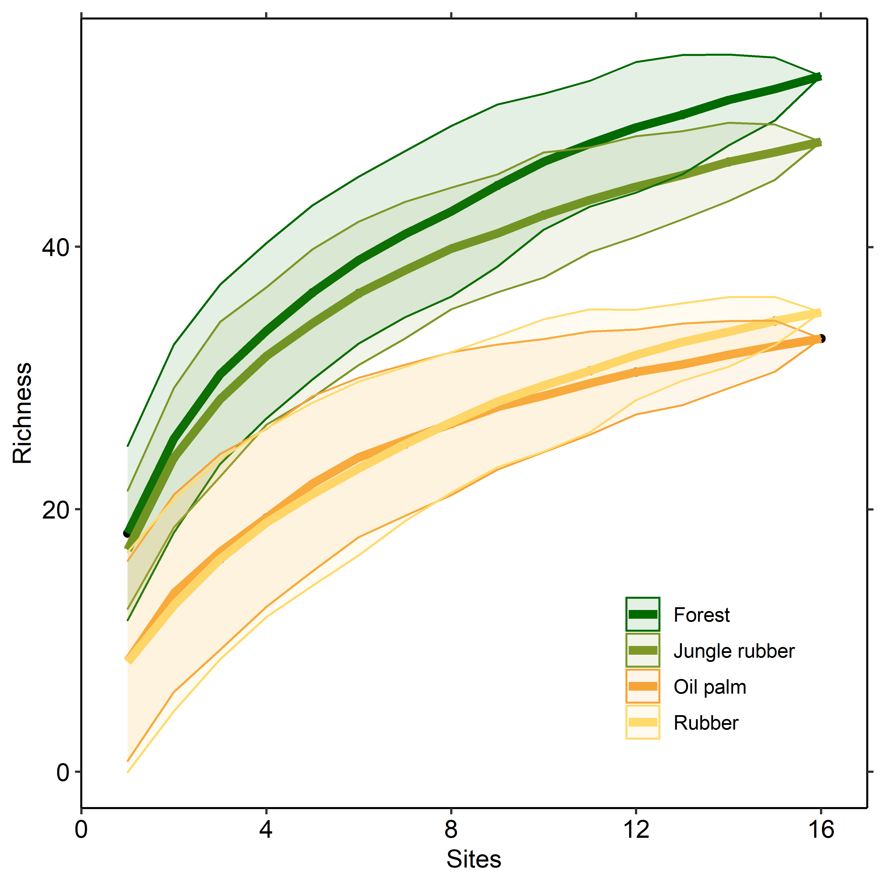


#### Figure S4. Location of the 32 study plots in two landscapes near Bukit Duabelas National Park and Harapan Rainforest in Jambi province, Sumatra, Indonesia (from [24]).


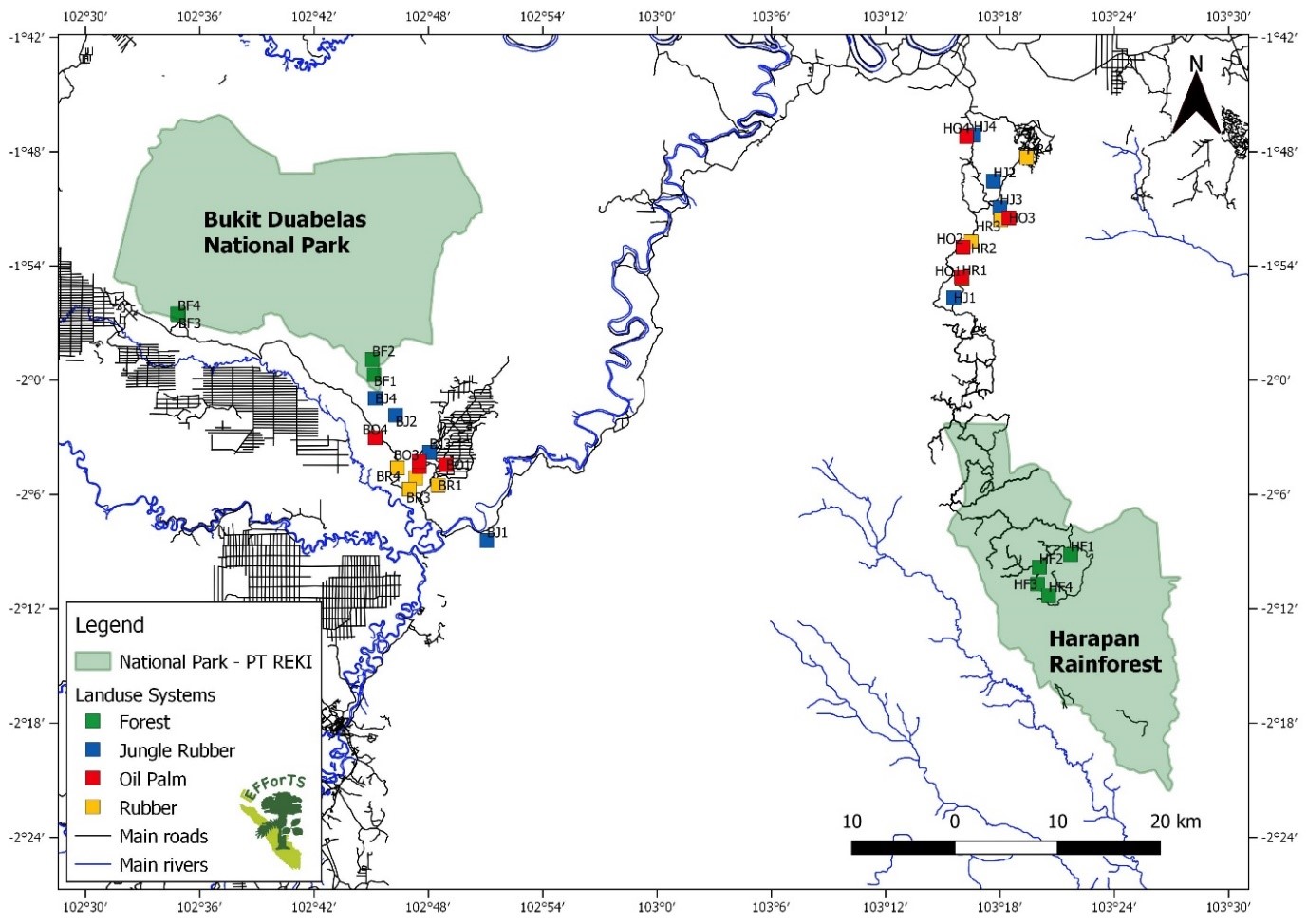


#### Figure S5. Overview of the investigated land-use systems: Lowland rainforest (A), jungle rubber (B), rubber plantation (C) and oil palm plantation (D). Photos by Jochen Drescher.

####
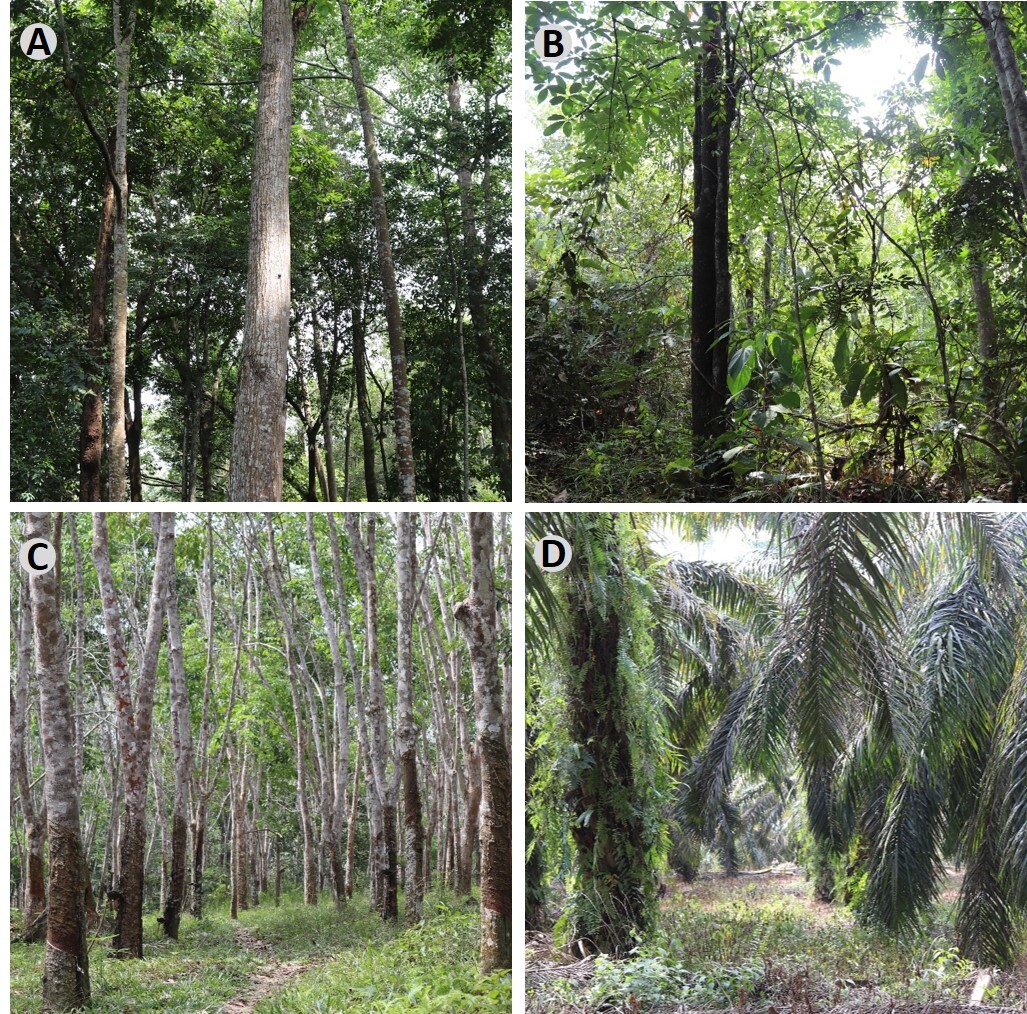

Supplement: Supplementary file 1 — Additional file 1: Figure S1. Species rank-abundance curves of arboreal Collembola in the four land-use systems studied (rainforest, jungle rubber, rubber plantation, oil palm plantation). Table S1. Relative abundance and rank of Collembola species in the four land-use systems (rainforest, jungle rubber, rubber plantation, oil palm plantation) in Jambi Province, Sumatra, Indonesia. Figure S2. Venn diagrams of shared and exclusive Collembola species in the four land-use systems (rainforest, jungle rubber, rubber plantation, oil palm plantation) (A), two landscapes (Bukit Duabelas, Harapan) (B) and two seasons (dry, rainy) (C). Figure S3. Species accumulation curves showing accumulation rates of new species in the studied four land-use systems (rainforest, jungle rubber, rubber plantation, oil palm plantation) in Jambi Province, Sumatra, Indonesia. Figure S4. Location of the 32 study plots in two landscapes near Bukit Duabelas National Park and Harapan Rainforest in Jambi Province, Sumatra, Indonesia (from [24]). Figure S5. Overview of the investigated land-use systems: Lowland rainforest (A), jungle rubber (B), rubber plantation (C) and oil palm plantation (D). Photos by Jochen Drescher. [file 12862_2022_2095_MOESM1_ESM.docx]
